# Supplementary material for: Co3O4 Nanoneedle Array Grown on Carbon Fiber Paper for Air Cathodes towards Flexible and Rechargeable Zn–Air Batteries
Source: Nanomaterials (Basel). 2021 Dec 7;11(12):3321. doi: 10.3390/nano11123321 (PMC8706223; doi:10.3390/nano11123321)
Supplement: Supplementary file 1 [file nanomaterials-11-03321-s001.zip › nanomaterials-1474622-supplementary.pdf]

Supplementary Materials

Co_3O_4 Nanoneedle Array Grown on Carbon Fiber Paper for Air Cathodes towards Flexible and Rechargeable Zn–Air Batteries

Ziyuan Li, Wenjia Han, Peng Jia, Xia Li, Yifei Jiang * and Qijun Ding *

State Key Laboratory of Biobased Material and Green Papermaking, Qilu University of Technology, Shandong Academy of Sciences, Jinan 250353, China; ziyuanli97@126.com (Z.L.); hwj200506@163.com (W.H.); milai19871219@163.com (P.J.); sqlixia126@126.com (X.L.)

* Correspondence: jiangyf@qlu.edu.cn (Y.J.); xianshengding@qlu.edu.cn (Q.D.)

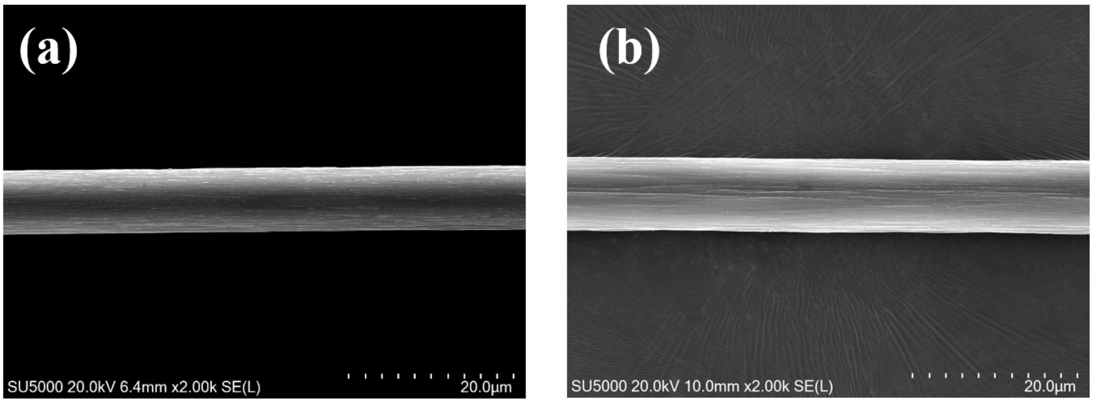

Figure S1. SEM images (**a**) CF, (**b**) m-CF.

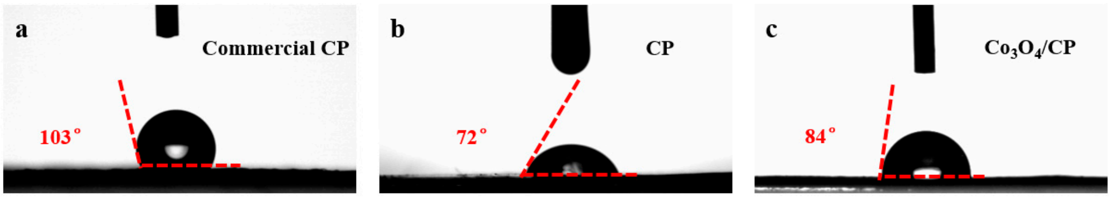

Figure S2. Contact angle image of (**a**) commercial CP, (**b**) CP and (**c**) Co_3O_4/CP.

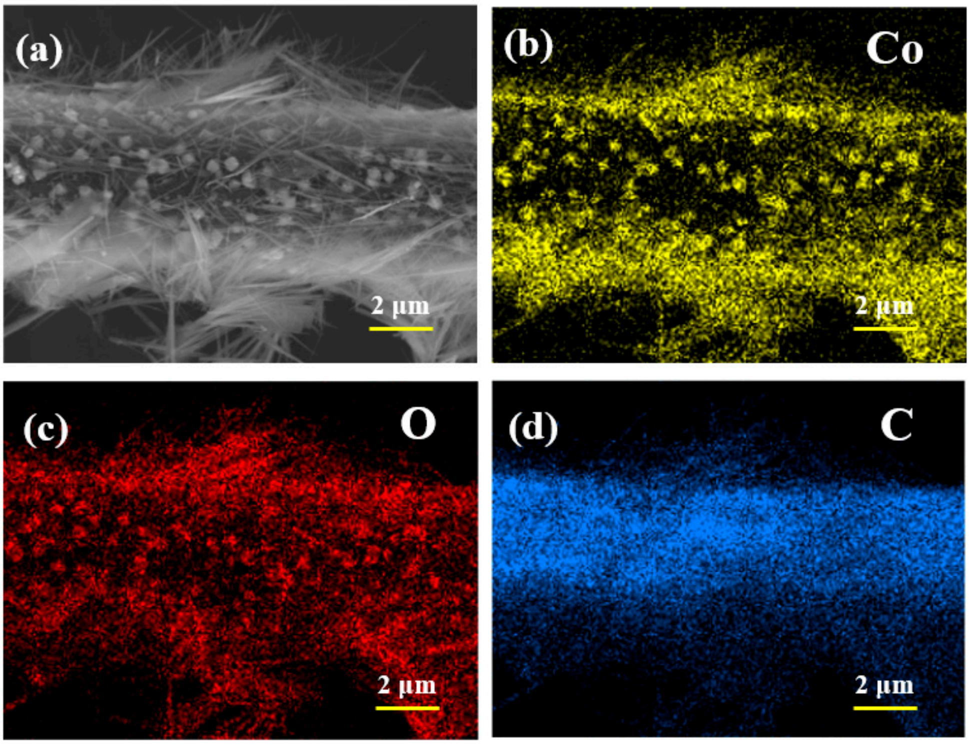

Figure S3. (**a**) SEM image of $Co(OH)_2$/m-CF and the corresponding EDS elemental mapping images of (**b**) Co, (**c**) O and (**d**) C.

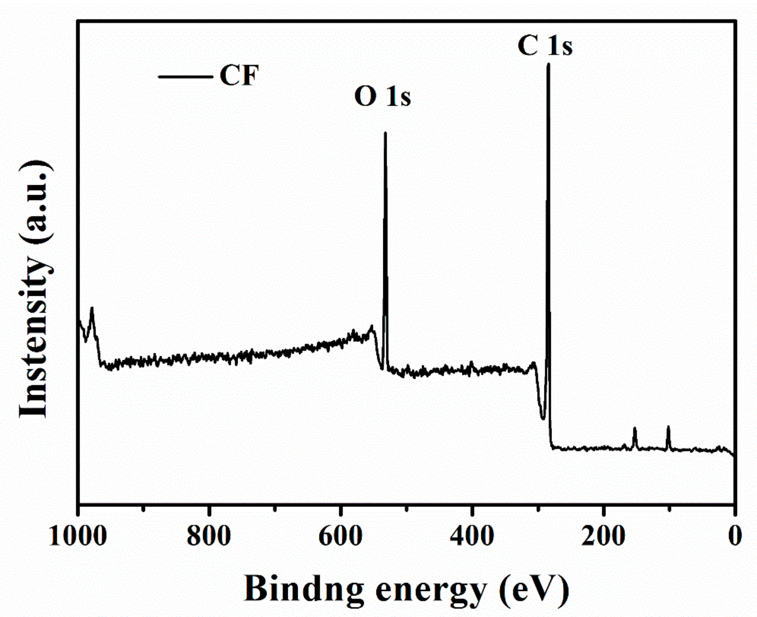

Figure S4. Survey XPS spectrum of m-CFs.

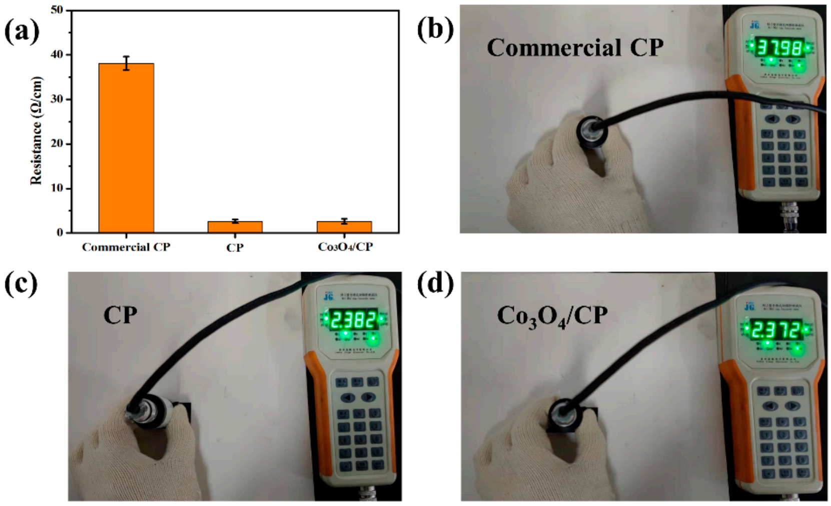

Figure S5. (**a**) various cathode conductivity data graphs; and actual detection images of (**b**) commercial CP, (**c**) CP, (**d**) Co_3O_4/CP.

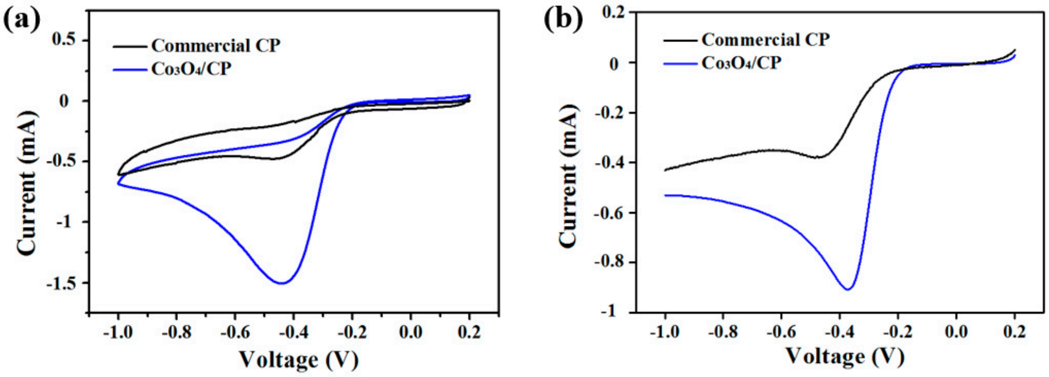

Figure S6. ORR performances of the commercial CP and Co_3O_4/CP: (**a**) CV curves; (**b**) LSV curves.

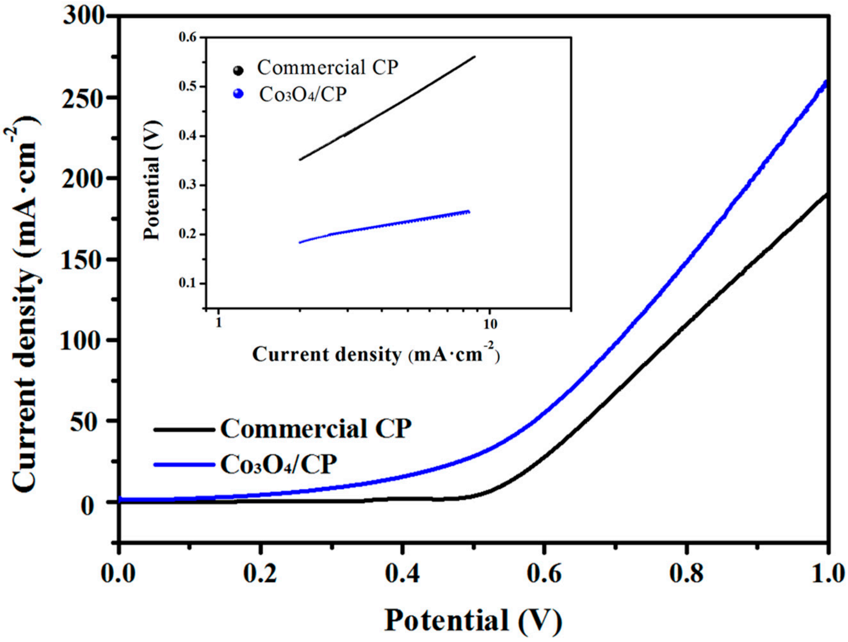

Figure S7. OER performances of the commercial CP and Co_3O_4/CP.

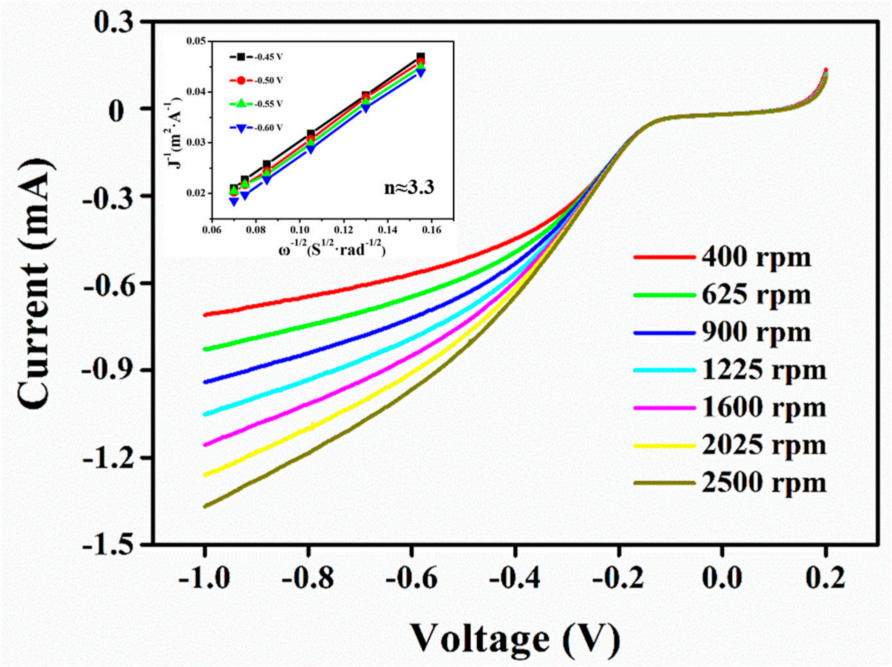

Figure S8. ORR polarization curves of Co_3O_4/CFP at different rotation speeds in O_2-saturated 0.1 M KOH solution.

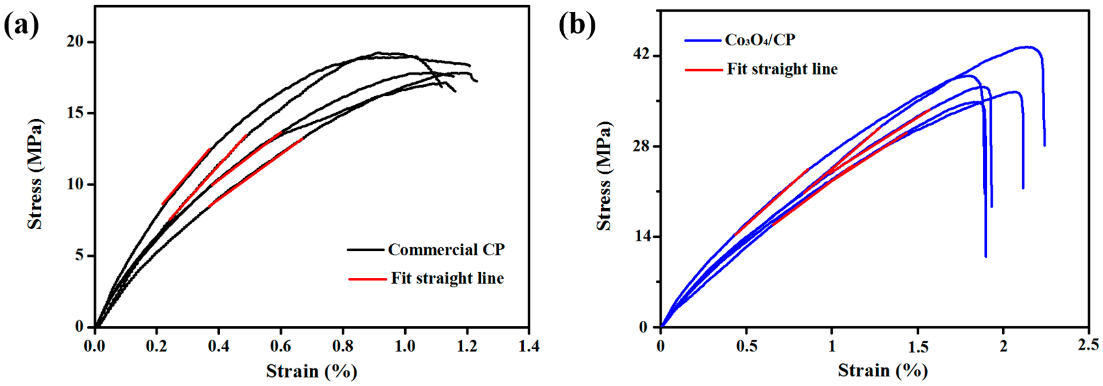

Figure S9. Repeated tensile test data of (**a**) commercial CP, (**b**) Co_3O_4/CP.

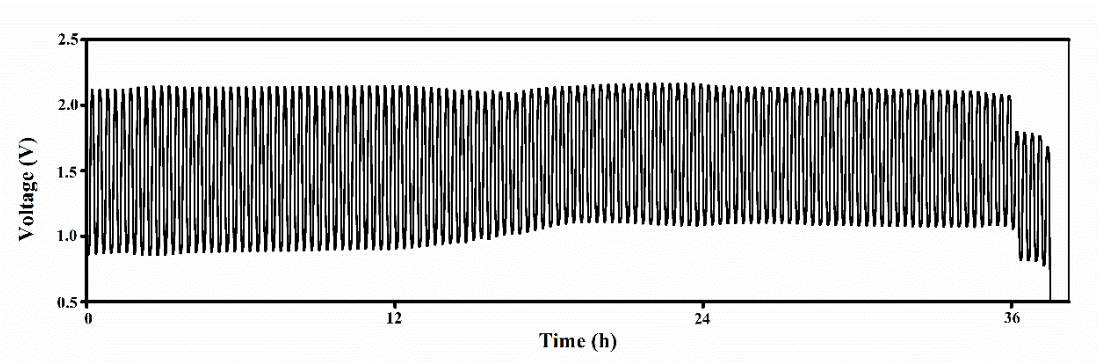

Figure S10. GCD cycle curves of the flexible zinc-air battery assembled with commercial CP.

Table S1. The weight percentage of elements in Co_3O_4/CP air cathode.

**Element**	**C**	**O**	**Co**
**Percentage (wt.%)**	70.47	15.99	13.54

Table S2. A summary of the battery performance of ZAB based on carbon fiber-based air electrodes in the literature.

**Air Electrodes**	**Electrolyte**	**Open Circuit Potential (V)**	**Discharge Capacity (mAh·cm^{-2})**	**Initial Round-Trip Efficiency**	**Cycling Stability (Time for per Cycle, Current Density)**	**Flexibility**	**Ref.**
Co_3O_4/CP	Gel PVA-KOH	1.34	67.4	60.4%	60 h (20 min, 2 mA cm^{-2})	Fully flexible	This work
Commercial Co_3O_4/ carbon cloth	Gel PVA-SiO_2-KOH	2.54 (Two batteries in series)	26.1	63.2%	48 h (20 min, 3 mA cm^{-3})	Fully flexible	[1]
Co_3O_4/ carbon cloth	Hydrogel electrolyte	1.33	5.4	52.8%	10 h (20 min, 2 mA cm^{-2})	Fully flexible	[2]
FeCo composite with N, S dual-doped carbon matrix	Solution 6M KOH	1.45	744 mAh/g	~60%	150 h (20 min, 3 mA cm^{-2})	-	[3]
N-Co_3O_4/ carbon cloth	Solution 6M KOH	1.576	–	61.1%	400 h (20 min, 15 mA cm^{-2})	-	[4]
N-Co_3O_4/ carbon cloth	Gel PVA-KOH	1.349	-	~55%	20 h (80 min, 30 mA cm^{-3})	Fully flexible	[4]

References

[1] Guan Q, Li Y, Bi X, Yang J, Zhou J, Li X, et al. Dendrite-Free Flexible Fiber-Shaped Zn Battery with Long Cycle Life in Water and Air. Advanced Energy Materials, 9 (2019) 1901434.

[2] Chen X, Liu B, Zhong C, Liu Z, Liu J, Ma L, et al. Ultrathin Co_3O_4 layers with large contact area on carbon fibers as high-performance electrode for flexible zinc-air battery integrated with flexible display. Advanced Energy Materials, 7 (2017) 1700779.

[3] Chang, S, Hui Z, and Zhang Z. FeCo alloy/N, S dual-doped carbon composite as a high-performance bifunctional catalyst in an advanced rechargeable zinc-air battery. Journal of Energy Chemistry, 56 (2021) 64-71.

[4] Wang X, Liao Z, Fu Y, Neumann C, Turchanin A, Nam G, Feng X. Confined growth of porous nitrogen-doped cobalt oxide nanoarrays as bifunctional oxygen electrocatalysts for rechargeable zinc-air batteries. Energy Storage Materials, 26 (2020) 157-164.
